# Modeling environmental contamination in hospital single‐ and four‐bed rooms

**DOI:** 10.1111/ina.12186

**Published:** 2015-03-04

**Authors:** M‐F. King, C. J. Noakes, P. A. Sleigh

**Affiliations:** ^1^ School of Civil EngineeringPathogen Control Engineering InstituteUniversity of LeedsLeedsUK

**Keywords:** HCAI, Indirect transmission, Hand contamination, CFD, Markov chain, Bioaerosol

## Abstract

Aerial dispersion of pathogens is recognized as a potential transmission route for hospital acquired infections; however, little is known about the link between healthcare worker (HCW) contacts’ with contaminated surfaces, the transmission of infections and hospital room design. We combine computational fluid dynamics (CFD) simulations of bioaerosol deposition with a validated probabilistic HCW–surface contact model to estimate the relative quantity of pathogens accrued on hands during six types of care procedures in two room types. Results demonstrate that care type is most influential (*P *<* *0.001), followed by the number of surface contacts (*P* < 0.001) and the distribution of surface pathogens (*P* = 0.05). Highest hand contamination was predicted during *Personal care* despite the highest levels of hand hygiene. Ventilation rates of 6 ac/h vs. 4 ac/h showed only minor reductions in predicted hand colonization. Pathogens accrued on hands decreased monotonically after patient care in single rooms due to the physical barrier of bioaerosol transmission between rooms and subsequent hand sanitation. Conversely, contamination was predicted to increase during contact with patients in four‐bed rooms due to spatial spread of pathogens. Location of the infectious patient with respect to ventilation played a key role in determining pathogen loadings (*P* = 0.05).

**Practical Implications**

We present the first quantitative model predicting the surface contacts by HCW and the subsequent accretion of pathogenic material as they perform standard patient care. This model indicates that single rooms may significantly reduce the risk of cross‐contamination due to indirect infection transmission. Not all care types pose the same risks to patients, and *housekeeping* performed by HCWs may be an important contribution in the transmission of pathogens between patients. Ventilation rates and positioning of infectious patients within four‐bed rooms can mitigate the accretion of pathogens, whereby reducing the risk of missed hand hygiene opportunities. The model provides a tool to quantitatively evaluate the influence of hospital room design on infection risk.

## Introduction

Risk of healthcare‐acquired infections (HCAI) is omnipresent in healthcare facilities worldwide, and understanding transmission routes is key to effective control. While the transmission routes for some diseases are well documented, the precise mode of transmission is uncertain for many infections, particularly for those pathogens that cause HCAI. Although it is probable that the majority of transmission occurs via a contact route (Sax et al., 2009), there is increasing recognition that the hospital environment plays an important role.

Evidence suggests that at least 20% of HCAIs potentially could have arisen from an environmental reservoir (Harbarth et al., 2003) and several recent studies have highlighted the importance of surface contamination and indicated a causal link to subsequent patient infection (Bhalla et al., 2004a). However, there is currently little robust understanding as to how healthcare worker (HCW)–surface contacts and activities in the healthcare environment result in HCW or patient exposure to such pathogens. Studies of human behavior in the healthcare environment have largely acknowledged the lack of a comprehensive survey which collects the minutiae of hand‐to‐surface contacts (Duckro et al., 2005; Pittet et al., 1999; Smith et al., 2012). Major studies have however, highlighted that environmental contamination, through the deposition of bioaerosol on surfaces, cannot be underestimated in the contribution to fomite‐based infection transmission (Dancer, 2008; King et al., 2013; Otter et al., 2011; Rusin et al., 2002). Surfaces such as bed rails have been linked with harboring microorganisms that cause hospital infections, and many frequently used surfaces in hospitals have been found to sustain viable pathogens including staphylococci, enterococci (Duckro et al., 2005; Hayden et al., 2008; Pittet et al., 2006), and *Clostridium difficile* (Roberts et al., 2008). In addition, indirect infection transmission shows a distinct possibility of being exacerbated by incomplete or non‐existent surface cleaning (Bogusz et al., 2013). Pathogens have been also shown to accrue on HCW hands as they touch surfaces (Pittet et al., 1999), and there is evidence they can subsequently be transmitted to patients (Hayden et al., 2008).

Hospital designs are tending toward incorporating single rooms, based on the premise that single room enables 24/7 admissions without disruption to other patients, and the design will improve confidentiality for clinical discussion, examinations, and treatment (Chaudhury, 2005). In reviewing the literature, Chaudhury (2005) further report single rooms to promote a therapeutic environment that is patient and family centered increasing recovery and reducing infections and risks and enhancing the healthcare professional and patient relationship. This evidence is supported by Ulrich and Zimring (2008), when considering the NHS Estates perspectives and further purport that single rooms provide increased levels of protection and prevention from infection.

In addition, research has explored these issues in depth, where there is an increasing body of evidence which points toward a relationship between hospital room design and infection control (King et al., 2013; Pittet et al., 2006; Shih et al., 2007; Tian et al., 2006). Ventilation is well recognized as a control strategy for airborne infection (Zhang et al., 2009) and is recommended for diseases such as tuberculosis, measles, SARS, and more recently influenza (Yang et al., 2011). Aerial dispersion of bioaerosols has been associated with non‐respiratory pathogens in hospitals including methicillin‐resistant *Staphylococcus aureus* (MRSA) (Sherertz et al., 1996) and *C. difficile* (Roberts et al., 2008), and subsequent contamination of surfaces has been recognized as a potential transmission route for some infections (Bhalla et al., 2004b). However, research into the combined role of airborne dispersion, pathogen contamination of hospital room surfaces, and interaction with human behavior is still in its infancy (Zartarian et al., 2012). Furthermore, the influence of airflow patterns and ward design on the risk of this combined transmission route is not well understood. Single‐bed rooms are widely advocated over four‐bed patient environments for their infection control potential, yet there are little data to allow quantification of the touted benefits (Ulrich and Zimring, 2008).

This research focuses on the combined interaction between deposition of airborne microorganisms, room design, and human behavior by considering the question: Are single‐bed patient rooms more effective than their four‐bed counterparts at reducing the risk of infection from environmental contamination? The study compares hospital single‐ and four‐bed room environments by modeling likely contamination on the hands of healthcare workers resulting from pathogens released from an aerosol source and deposited on surfaces. The model combines computational fluid dynamics (CFD) simulations of room airflow and particle deposition (King et al., 2013) data from HCW hand–surface contact patterns established through a hospital observational study and a dermal pathogen accretion model to simulate contamination of HCW hands. The resulting probabilistic pathogen accretion model (PAM) is used to compare the effect of room layout and ventilation rate on HCW hand contamination during six different types of patient care.

## Methodology

### Patient rooms

**Figure 1:**
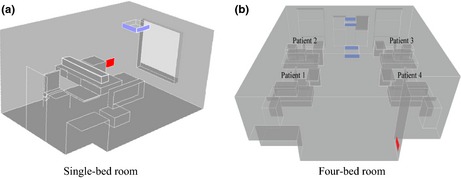
Typical room layouts from HBN04‐01, as used in CFD simulations, showing simplified furniture arrangement, ceiling mounted supply and wall mounted extract. (a) single room and (b) four bed room

Two room scenarios are considered: a typical hospital single room and a standard four‐bed room. Both room layouts are based on the UK Department of Health standard, HBN04‐01(Department of Health Estates and Facilities Division, 2008), and computer aided design models are illustrated in Figure 1.

Rooms are assumed to be fully occupied, with one infectious patient in each scenario. In both rooms, modeling is conducted with the ventilation at 6 air changes per hour (ac/h), as per the UK Department of Health guidance, HTM03‐01 (Department of Health, 2007) and at a reduced rate of 4 ac/h. In the case of the four‐bed ward (*cases 3–10*), the relative influence of ventilation design was also considered through changing the location of the infectious patient. All cases together with a brief description are shown in Table 1.

**Table 1:** Case study scenarios

Case N°	Room type	Room dimensions	ac/h	Infectious patient
1	Single‐bed	4.7 × 3.8 × 2.8 m	4	Patient's head
2	Single‐bed		6	Patient's head
3	Four‐bed	6.8 × 7.6 × 2.8 m	4	Patient 1
4	Four‐bed		4	Patient 2
5	Four‐bed		4	Patient 3
6	Four‐bed		4	Patient 4
7	Four‐bed		6	Patient 1
8	Four‐bed		6	Patient 2
9	Four‐bed		6	Patient 3
10	Four‐bed		6	Patient 4

### Environmental contamination

Computational fluid dynamics (CFD) simulations were carried out to predict the likely spatial deposition pattern resulting from bioaerosols released from a quiescent patient in the single‐ and four‐bed room. The CFD methodology is detailed below and has previously been shown to compare well to experimental measurements of bioaerosol deposition in chamber studies (King et al., 2013). In this previous study hospital, single‐bed and two‐bed rooms were replicated inside an aerobiology chamber at the University of Leeds (4.2 ×3.2 × 2.2 m) with steady ventilation at 6 ACH. *Staphylococcus aureus* was aerosolized by a Collison Nebuliser (CN 25; BGI Inc., Waltham, MA, USA) (mean diameter 2.5 *μ*m) and released into the room for 30 min at the head of a supine heated mannequin. Deposition of bacterial colonies was quantified on key surfaces by means of nine agar‐filled petri dishes per surface CFD simulations conducted in Fluent v.13 (ANSYS, Canonsburg, PA, USA) and following the methodology outlined below were used to model deposition of 2.5 *μ*m particles released at the same location and with the same geometry as the experimental scenarios. Normalized concentrations on surfaces were seen to compare well between the experiments and CFD model, giving confidence that the CFD modeling approach can predict a realistic spatial deposition pattern in a ventilated room.

In the current study, steady‐state airflow simulations were carried out using Fluent v.13 (ANSYS) for the model hospital room geometries shown in Figure 1. Meshing, carried out in ANSYS Workbench v13, was fully hexahedral. Grid density and construction has been shown to heavily influence flow results (Roache, 1997); therefore, a mesh independence study was based on the formulation set out in King et al. (2013). For this three hexahedral, grids with nominal cell sizes of 50, 25, and 20 mm were compared. Velocity variation was found to differ less than 5% between the 25 and 20 mm meshes at selected points within the bulk flow. A maximum cell volume of 1.5625 × 10^−5^ m^−3^ was used within the bulk domain, and 1 × 10^−6 ^m was used 10 cm away from all horizontal surfaces. Final cell count for all simulations was approximately 4 and 8 million volumes for the single‐ and four‐bed rooms, respectively.

Mechanical ventilation was modeled through a simplified four‐way ceiling supply diffuser (Zhang et al., 2009) with velocity boundary conditions specified to give the air change rates in Table 1. Air was assumed to be extracted via a wall‐mounted grille, with a pressure boundary condition of −10 Pa. Turbulence was modeled with a Reynolds’ Stress (RSM) closure model and standard wall functions; this model was shown in the previous study to compare better with experimental bioaerosol deposition data than the more commonly used k‐epsilon model (King et al., 2013). Human patients lying supine on the beds were represented through simplified blocks emitting a heat flux of 56 W/m^2^. Air entering the room was at 20°C and all walls were adiabatic. Bioaerosols were modeled using a Lagrangian particle tracking method with discrete random walk. In each case, 10,000 2.5 *μ*m diameter inert particles were released via a volume source located 10 cm above the patient's head and given an inlet velocity of 1 m/s in the positive vertical direction to represent exhalation.

Nine surfaces of interest were defined within the CFD model, as indicated in Table 2, and the modeled particle concentration was determined on the surfaces for each scenario in Table 1. These surfaces were based on those monitored during the observation study outlined below.

**Table 2:** Surface categories and modeled surfaces in CFD geometries

Equipment	Patient	Near patient	Far patient	Hygiene areas
Location on bed wall (1 × 0.4 m)	Mannequin	Bed	Window	Sink
		Chair	Workstation	Towel dispenser
		Tray		
		Bedside table		

CFD simulations were carried out on a University of Leeds’ high‐performance facility (ARC1 with 672 AMD cores and 128 GB memory), were considered converged when residuals for continuity and all other variables dropped below 1 × 10^−4^, and remained below this for at least 100 iterations. All variables are scaled with respect to the sum of the errors in all cells. In addition, continuity is scaled with respect to the largest absolute value within the first five iterations and so can be considered normalized (ANSYS 2010).

### HCW–surface contacts

The likelihood of healthcare workers having contact with surfaces during patient care was developed from surface contact data accrued during an observational study carried out at a National Health Service (NHS) single‐bed accommodation hospital ward during the first quarter of 2012. Healthcare workers were observed during episodes of care categorized into six areas described in Table 3, following standardized categories set out in Pittet et al. (1999) and Dancer et al. (2010).

**Table 3:** Six care types with examples of procedures within each type

Direct care	Housekeeping	Mealtimes	Medication rounds	Miscellaneous	Personal care^a^
Blood pressure measurement	Equipment cleaning	Distribution of meals	Distributing medication	Call bell request	Toileting
Weighing patients	Cleaning patient surfaces		Injections	Response to pressure mattress alarms	Patient changing
Blood sugar saturation					

a Note that limited observations were made for personal care episodes for patient privacy reasons.

**Figure 2:**
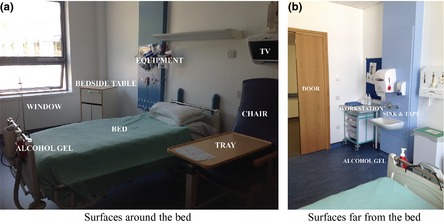
Typical location of surfaces in the single room which were monitored for HCW contacts. (a) room surfaces close to the bed and (b) surfaces close to the door

In each observed care episode, hand‐to‐surface contacts with surfaces set out in Table 4 and the sequence with which they happened were recorded. Typical locations of these surfaces are shown in Figure 2. The occurrence and type of hand hygiene after each care episode was also recorded. Over 400 individual care episodes were observed.

**Table 4:** Surfaces within each surface category during the monitoring study

Surface category				
Equipment	Patient	Near patient	Far patient	Hygiene areas
IV stand	Clothing	Bedrail	Window	Alcohol gel
Hoist		Bedding	Curtain	Soap dispenser
BP cuff/stand		Tray	Light switch	Tap
Notes trolley		TV	Chart	Sink
Medication trolley		Chair	Door/handles	Paper towel dispenser
		Bedside table		

Data from the observations were used to generate representative contact patterns to model the likely sequence of surface contacts by a HCW in a patient room. HCW were found to touch surfaces in a non‐random sequential or directed manner, insofar that moving from one surface category to another has a higher probability than a transition to somewhere else. By assigning each surface category a numerical value from 1 to 5, where *Equipment *= 1, *Patient *= 2, *Hygiene areas *= 3, *Near‐bed surfaces *= 4, and *Far‐bed surfaces *= 5, the movement of the HCW between surfaces can be represented by means of a weighted probability.

The movement of HCW between surfaces is modeled using a Markov chain approach (Allen, 2008). Using the defined weighted probabilities, the movement of a HCW between surfaces can be simulated based on the property that, given the present state, the future and past states are independent. This is termed the Markov Property:

(1) $$P(X_{i}=x_{i}|X_{j}=x_{j}).$$

Given the five surface categories, the probabilities can be expressed in a transition matrix (*P*) indicating the maximum likelihood of directed contact between any pair of surfaces. For example, if the HCW is currently touching surface category 1, p_12_ is the probability that they will then touch surface category 2. This probability is derived from the observed data by counting the occurrences of the transition 1→2 for each HCW in the observational study and dividing by the total number of surface transitions throughout their episode of care. Thus, ${\hat{P}}_{ij}=\frac{x_{ij}}{\sum_{m}x_{ik}}$, where *m* = 1…5 states, and indices *i*,* j*, and *k* are dummy indices to indicate summation.

The observed data enabled surface contact transition matrices to be created for single‐patient rooms, but to compare single‐ and four‐bed rooms, HCW behavior in the four‐bed room must be modeled. This was undertaken by assuming that care approaches were similar in the two room types and adjusting the transition probabilities based on other published information. Smith et al. (2012) conducted an observational study of HCWs in a four‐bed room applying the same surface category criteria as used here. However, they note that patient charts become near‐bed objects as these are often at the foot of the patient's bed rather the far side of the room; in our single‐bed rooms, chart positions were modeled as a far‐bed surfaces as the chart was located on the workstation (Figure 2) in the observation study. Therefore, this adjustment is made to the probability densities derived from the healthcare observations. A Wilcoxon rank‐sum nonparametric test comparing Smith et al.'s (2012) data to the current study shows that no statistical difference exists at the 5% level (*P* = 0.068), highlighting that the only difference in HCW behavior between single‐ and four‐bed rooms is the positioning of patient charts. Consequently, this demonstrates the similarity between nurse behavior in single‐ and four‐bed rooms. All four‐bed simulations use this adjusted behavior in line with Smith et al. (2012).

### Pathogen accretion model

Colonization of HCW's hands through contact with a contaminated surface is considered to be a dynamic process in which multiple factors may vary. The number of microorganism colony forming units, *Y* (cfu), accrued on HCWs hands from environmental or patient surface contacts during patient care can be shown via sensitivity analysis to depend primarily on six variables (Pessoa‐Silva et al., 2004).

(2) Y=Y(n,A,V,λ,β,h).

#### The number of surfaces touched, n

Each type of care shows a variable pattern of surface contact frequencies and sequences. Probabilistic distributions for these were obtained from the observational study as outlined above.

#### Surface contamination, V (cfu/cm^2^)

The concentration present on each room surface is dependent on the scenario in question. These are obtained from sampling the lognormal distribution based on the CFD simulations for each scenario, as described above. It must be highlighted that this study considers no microbial die‐off due to environmental stress as this is a time‐dependent phenomenon. Therefore, *V* is assumed to be proportional to the amount released initially in each case. The parameter *V* is regarded as the initial condition for the PAM model simulations, with the surfaces already contaminated at the point the HCW is assumed to enter the room. In addition, if each particle is treated as one cfu, the resulting deposition in cfu/cm^2^ is comparable to actual hospital values (Mulvey et al., 2011).

#### Hand–surface contact area, A (cm^2^)

This is assumed to exhibit a continuous distribution based on experimental values from Brouwer et al. (1999). They show that this can be modeled as a lognormal distribution with an arithmetic mean of 7 cm^2^ and standard deviation of 1.9 cm^2^ [corresponding to lnN~(0.84,1.3)]. For each surface contact, it is assumed that either hand may be in contact with the surface and no distinction is made for right‐ or left‐handedness.

#### Surface‐to‐Hand Transfer efficiency, **λ**

**Figure 3:**
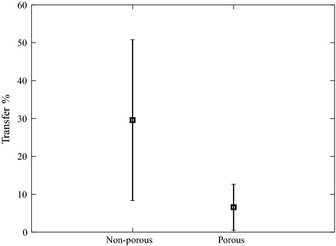
Surface‐to‐hand transfer efficiency (*λ*). Data derived from Rusin et al. (2002)

It is reasonable to assume that not all of the pathogens in contact with the area of skin touching the surface are transferred to the hands. Transfer efficiency, which represents the percentage of surface contaminant transferred to the hand during a contact event, has been shown to be one of the most important parameters when modeling dermal exposure (Xue et al., 2006). Unfortunately, it is one of the most troublesome to accurately measure (Beamer et al., 2009). Transfer efficiency could be a function of multiple ambient parameters such as surface physiology, contact frequency, duration and pressure, concentration of transferrable material on surface, temperature, and humidity. Rusin et al. (2002) conclude from experimental studies that the transfer efficiency from surfaces to hands also varies between microorganism species. In three separate experiments, they reported the transfer of Gram‐positive bacteria, Gram‐negative bacteria, and bacteriophage to be 38.5%, 65.8%, and 27.6–40%, respectively, from non‐porous surfaces. However, they indicate the transfer rate reduces to below 10% when porous fomites are evaluated. As there are insufficient data available in the literature to model the influence of specific parameters, the model employs the distribution developed by Rusin et al. (2002), as shown in Figure 3, to represent the variability in transfer efficiency.

#### Hand‐to‐surface transfer efficiency, B

A significant quantity of material residing on hands may also be transmitted to the surface during contact (Otter et al., 2013). Montville and Schaffner (2003) demonstrate that a difference exists between the direction of transfer, with statistically significantly lower transmission shown during transfer from hands to fomites than vice versa. They also highlight the influence of inoculum size on the efficiency of transfer, showing that when high levels are used, transfer rates are more accurately characterized (Montville and Schaffner, 2011). Surface‐to‐hand transfer efficiencies (*β*) are sampled from Monteville et al.'s empirical data. Therefore, during hand‐to‐surface contact, a proportion of pathogens already acquired by the HCW may be deposited from the hand onto the surface (Rusin et al., 2002). Random sampling is conducted from data in Montville and Schaffner (2003).

#### Antisepsis efficacy, h (%)

This refers to the efficiency of reducing microbial contamination of HCWs’ hands after performing one of three types of hand hygiene: hand washing with either bland or medicated/antibacterial soap, removal of non‐surgical gloves, or dry rubbing with a waterless alcohol agent (minimum 61% ethanol by volume). Here, hand hygiene efficacy is based on data from published studies where significant sample sizes are available (Girou et al., 2002; Montville and Schaffner, 2003; Sickbert‐Bennett et al., 2005). Microorganism type has been shown to exert a difference on hand hygiene efficacy, which is likely to be due to their individual adherence properties (Boks et al., 2008). Particular difference is noticed between bacteria and viruses (or bacteriophage), where removal of the latter is often an order of magnitude lower. Nevertheless, bacteria have the primary concern of many infection control teams, where MRSA or *C. difficile* often rank highest on the prevention list. Consequently, the model is non‐organism specific, whereby resident or background microflora is ignored. Whether or not hand antisepsis actually occurs following an episode of patient care is based on the probabilities derived from the observation study and depends on care type.

Following a variance based sensitivity analysis using total effect Sobol indices (Nossent et al., 2011), the function shown in Equation (2) can be used to model pathogen accretion (*Y*) by means of a recurrence relationship given in Equation (3). This represents the hand pathogen loading at any time‐step after contact with any one surface.

(3) $$Y_{i}=\lambda_{i}V_{i}A_{i}+\beta_{i}Y_{i-1},$$

where *i = *1…*n* is the surface contact count. For purposes of remaining succinct, let *σ* = *λAV* then assuming that the transfer of pathogens to the surface from the HCW occurs sequentially and is independent of the surface loading then *Y* can be described as follows:

(4) $$\begin{array}{c} Y_{i}=\sigma_{i}+\beta_{i}*\left(\sigma_{i-1}+\beta_{i}*\left(\sigma_{i-2}+\cdots \right)\right)\\ =\sigma_{i}+\beta_{i}\sigma_{i-1}+\beta_{i}\beta_{i-1}\sigma_{i-2}+\cdots \\ =\sum_{j=0}^{i}\left(\prod_{k=j+1}^{i}\beta_{k}\sigma_{j}\right). \end{array}$$

HCW touching surfaces during an episode of patient care forms the driving force within this model. The model was programed using Matlab (MathWorks, Natick, MA, USA). For each scenario, a Monte Carlo simulation of 1,000 HCW care episodes was conducted for each care type, modeling the mechanics of pathogen transfer based on the input parameters set out above.

## Results and discussion

### Bioaerosol deposition

**Figure 4:**
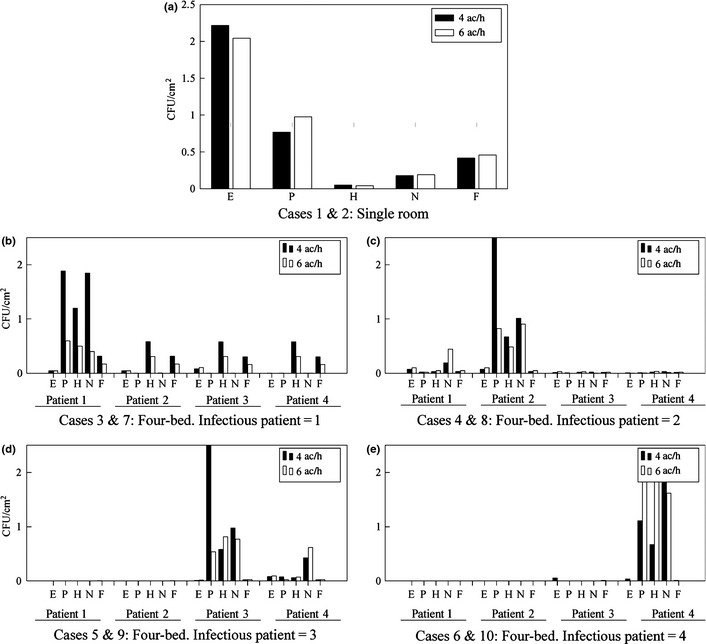
Predicted particle deposition (cfu/cm^2^) on horizontal surfaces within the single‐ and four‐bed accommodation for an initial release of 10,000 cfu. Comparison shown between 4 ac/h and 6 ac/h for each case. (a) case 1 & 2 in the single room, (b) cases 3 & 7 in the four‐bed room where the infectious patient is in position 1, (c) cases 4 & 8 in the four‐bed room where the infectious patient is in position 2, (d) cases 5 & 9 in the four‐bed room where the infectious patient is in position 3 and (e) cases 6 & 10 in the four‐bed room where the infectious patient is in position 4. E = Equipment, P = Patient, H = Hygiene areas, N = Near‐patient surfaces, F = Far‐patient surfaces

Surface deposition concentrations, cfu/cm^2^ predicted from the CFD simulations following the release of 10,000 cfu, can be seen in Figure 4. These show the breakdown of particle concentration for the five different surface categories and, in the four‐bed case, for each infectious patient position in turn (Figure 4b–e). Deposition in the single room (Figure 4a) appears to be mainly focused on the equipment area and the patient themselves, and the model predicts little difference in the deposition pattern between 4 and 6 ac/h. In the case of the four‐bed room, predicted surface concentrations are highest in the vicinity of the source patient in all cases. Surfaces opposite the source position demonstrate a sharp drop‐off; here, particles appeared to be maintained airborne and directed toward the extract. This is particularly the case when *patient 2* is the source (Figure 4c), where a seemingly dichotomous partition within the room can be observed, and few particles are deposited on *patients 3* and *4*. Equally when *patient 3* is the source (Figure 4d), negligible counts can be found in the vicinity of *patient 1 or patient 2*, and when patient 4, closest to the extract, is the source, there are very few counts in the vicinity of any of the other patients. However, the distribution when *patient 1* is the source (Figure 4b) is noticeably different with deposition predicted across the room.

Wong et al. (2010) suggest that as the air change rate increases, the deposition increases further from the source. A one‐way nonparametric Wilcoxon signed‐rank test does not uphold this conclusion here, and there is no clear difference between the two air change rates. However, a statistically significant difference is exhibited in the four‐bed room (*P* = 0.04) between surface concentrations for different infectious patients at the same air change rates.

### Surface contact sequences

**Figure 5:**
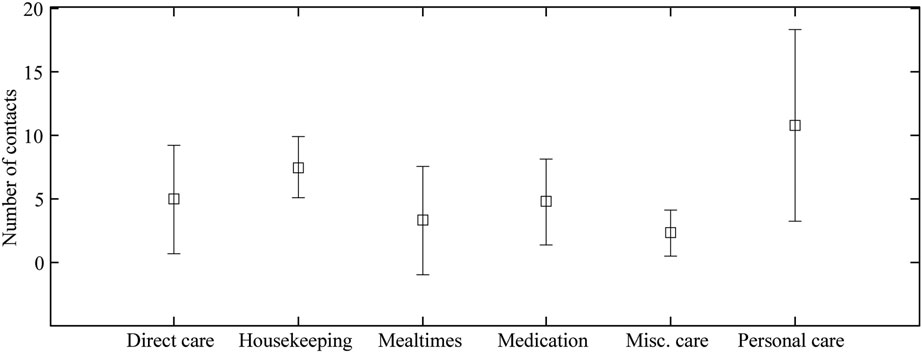
Mean and standard deviation of surface contacts observed and used in the model

Observational study data indicated the probability of contact with different surface categories (*P* = 0.04) along with hand hygiene method (*P* = 0.02) was dependant on care type. Statistical differences were also significant between the total number of surface contacts and the likelihood of hand hygiene being performed for different care types. It should be noted that this is not analogous to hand hygiene compliance as the research team are not able to pass judgment on what hygiene procedures should be followed for a particular care episode. Figure 5 shows the mean and standard deviation of surface contacts observed and used in the model:

Table 5 shows transition matrices $\hat{P_{ij}}$ for all care types derived from all observed HCW surface–contact sequences. Briefly recapping, the transition states are as follows: *1 = Equipment, 2 = Patient, 3 = Hygiene areas, 4 = Near‐bed surfaces,* and *5 = Far‐bed surfaces*.

**Table 5:**
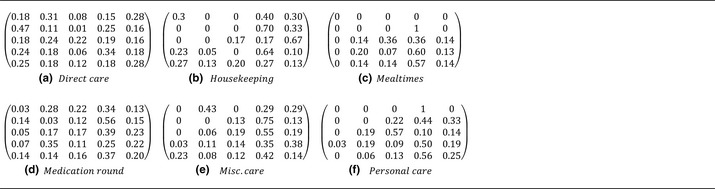
Transition matrices $\hat{P_{ij}}$, showing directed probabilities of moving from surface *i* to surface *j* for each type of care

**Figure 6:**
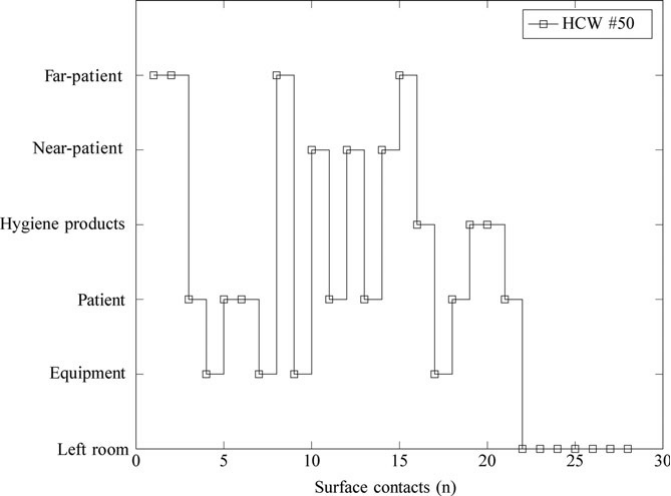
Example of surface contacts of HCW #50 performing an episode of *direct care*

Using these directed probabilities, Figure 6 shows a typical example sequence of surface contacts by a HCW during an episode of care in a single room by means of a stochastic simulation.

### Hand hygiene

**Figure 7:**
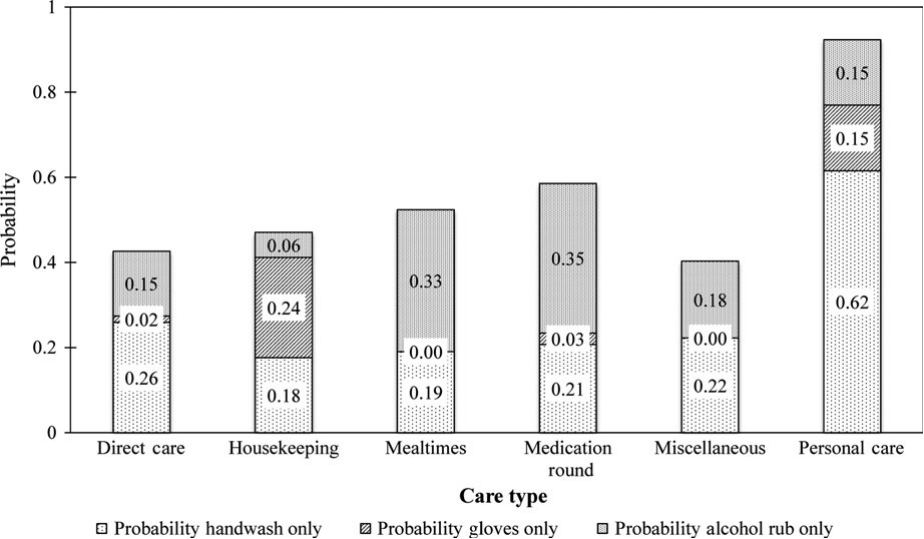
Cumulative probability of hand hygiene category subdivided by care type

The observational study data showed statistically significant differences in hand antisepsis choice between care types (*P* *<* 0.001), with some variation within each care type, as shown in Figure 6. Alcohol rub was used abundantly throughout all but *personal care*, where hand washing with soap and water predominated. Glove usage accounted for only 2% of observed episodes of care, half of which were during *housekeeping*. These probabilities are incorporated into the PAM model to account for the likelihood of HCWs undertaking some form of antisepsis after each episode of care (Figure 7).

### Model validation

Published data which measures contamination levels of HCWs’ hands are scarce. In particular, Pittet et al. (1999) is the only known published study that quantifies both cfu values along with the time spent in the room by the HCW. However, their methodology does not include surface swabbing and hence a specific value for surface contamination, *V*, is not known. Nevertheless, assuming that other variables such as HCW hand surface area, surface types, and nursing behavior are comparable between scenarios, it is reasonable to compare the distribution shape by means of a Kendall‐tau nonparametric test of the simulated cfu from PAM and the measured data by Pittet et al. (1999). Specifically, this is a measure of rank correlation or the similarity of the orderings of the data when ranked by each of the quantities. This approach is used for comparing the differences between individual observations with respect to each other to gain an insight into the overall data distribution.

Initial comparison shows a less‐than‐perfect fit (*P* ~ 0.2), and therefore, the data from both PAM and Pittet et al. were split into 4 groups corresponding to: lower quartile (≤25%), lower‐mid quartile (25% < *x* ≤ 50%), upper‐mid‐quartile (50% < *x* ≤ 75%), and upper quartile (75% < *x* ≤ 100%). Good comparison is shown particularly at lower (first quartile) values (*P* = 1 × 10^−3^), where a higher concentration of data exists. Both lower‐mid and upper‐mid quartiles compare well (*P* = 0.01 and *P* = 0.04). Higher values of cfus (upper quartile) become less frequent and the comparison is poorer (*P* = 0.12). The reader must note that detection levels by glove juice methods and subsequent culture techniques carried out by Pittet et al. (1999) have led to truncated data, which is likely the reason for less optimal comparison at this level. If these data were extrapolated, PAM potentially may approximate the behavior better in the upper quartiles (*P* ~ 0.02). However, pending greater sources of data, it is not unreasonable to conclude that at least through the first two quartiles or the first 50% of cfu colonization levels, PAM is a capable and realistic model.

### Application of PAM to single‐ and four‐bed rooms

#### Air change rate

Results from PAM are all normalized with respect to the mean contamination level on HCWs’ hands after *direct care* in the single room at 6 ac/h. This can be considered the ‘base case’ and enables comparison between rooms, ventilation, and care types.

**Figure 8:**
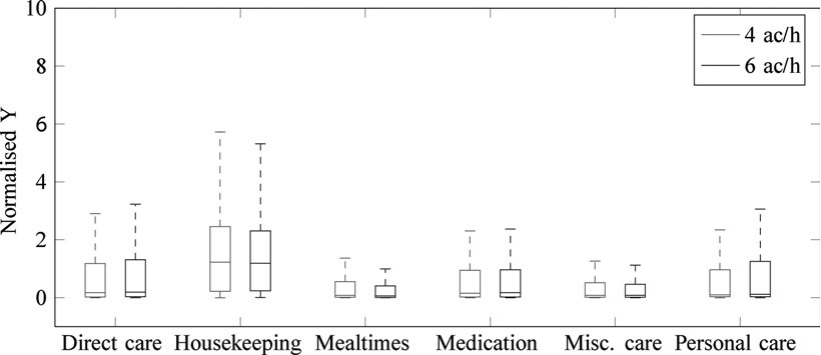
Boxplots showing predicted normalized cfu values on HCW hands for a single room before hand hygiene, comparing 4 and 6 ac/h

Figure 8 depicts predicted normalized cfu on HCW hands, *Y*, compared against the average of *direct care* for each subsequent type of care within the single room. Additionally, the comparison is made between a ventilation rate of 4 and 6 ac/h. Initially, there appears only to be a small reduction in hand contamination from 4 to 6 ac/h when comparing medians, and comparison of the upper quartile shows small differences depending on care type. However, comparison of extrema using a Kruskal–Wallis nonparametric test reveals that predicted hand contamination levels under a ventilation rate of 4 ac/h are consistently higher throughout (*P* < 0.05).

#### Room type

**Figure 9:**
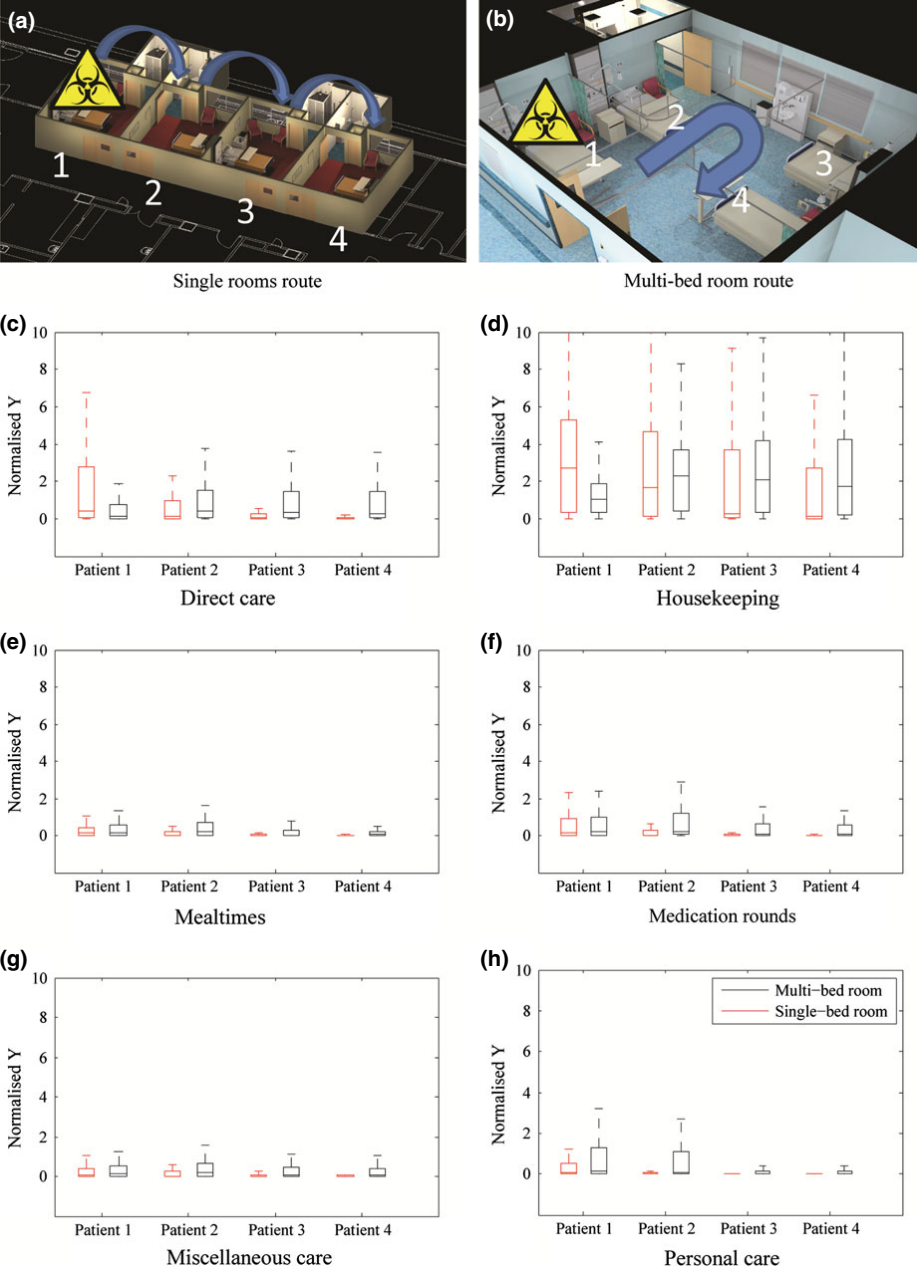
HCW route for all care types in the single‐ and multi‐bed rooms at 6 ac/h (a, b). Boxplots (outliers omitted) of cumulative normalized *Y* values after different episodes of care (c–h). Multi‐bed plots are an average of four episodes of care, varying the infectious patient location

In Figure 9, we consider two design options: four patients located in four single rooms or colocated in one‐four‐bed room. In each case, the simulation assumes that one patient is infectious and that the HCW provides care to all four patients in turn. The model assumes the worst case scenario where the first patient attended to is the infectious one (Figure 9a,b). All other parameters in the model, including hand washing are as set out in the methodology. Figure 9c–h shows the normalized contamination on HCW hands (Y values) for progression through the series of four patients in single‐ and four‐bed rooms with no airborne cross‐transmission between the rooms. The results show that cfu values are likely to be monotonically decreasing as the HCW progresses between patients in the single rooms. Environmental contamination due to the patient is only present in room 1, and hand antisepsis removes contamination in the majority of cases and minimizes further environmental contamination in subsequent rooms. In the case of the multi‐bed room, the results are quite different. Rather than an expected decrease due to hand hygiene, the aerial spread of microorganisms to neighboring bed bays allows for subsequent accretion of pathogenic material by the HCW regardless of hand hygiene. This accretion is also likely to happen without the HCW awareness; they may take additional precautions if they know that patient 1 is infectious, but are not likely to modify care for other patients in the same way.

## Discussion

The study conducted here demonstrates the potential for quantifying the effect of room design on potential infection risk by means of an indirect metric, namely hand contamination.

### Bioaerosol deposition patterns

CFD modeling of the application scenarios revealed that a ventilation rate of 6 ac/h showed little significant improvement over 4 ac/h on deposition quantities or spatial variation in three of the four considered scenarios in the four‐bed room. A decrease in spread locus was noticed particularly when the infectious patient was placed closest to the outlet vent, highlighting the greater downward momentum on particles exerted by comparatively higher local air velocities at 6 ac/h. This indicates that both the ventilation diffuser locations and ventilation rate may be important in influencing deposition as well as extraction of airborne microorganisms. It is recognized that exact cfu/cm^2^ values cannot be obtained via this method due to the absence of knowledge regarding the concentration of the infectious source. Here, we used a normalization metric to consider the relative spatial deposition. It is also important to note that the CFD simulations were steady‐state models of the airflow that did not account for movement of people and only considered the deposition of small (2.5 *μ*m) diameter particles. We made these assumptions here based on validation experiments for the CFD models (King et al., 2013). In reality, infectious particles will be released in a size distribution that will include smaller and larger particles which will change the spatial deposition patterns; larger particles are likely to increase deposition close to the patient. A CFD model incorporating a polydisperse particle release based on known particle size data from respiratory (Han et al., 2013; Xie, 2008) and environmental (Hathway et al., 2013) sources could enable an estimation of the resulting spatial contamination, which can then be applied in PAM. However, without experimental validation of the CFD model, these results would have to be treated with caution.

Ventilation method and transient flows due to activity are also factors. Under the HTM 03‐01UK guidance (Department of Health, 2007), minimum air change rates of 6ACH are specified, but how and if these are achieved can vary. Hospital ventilation strategies in the UK often incorporate both natural and mechanical ventilation methods. While verification of mechanical flow rates is largely feasible, in naturally ventilated environments, measurement is more challenging. Although the small number of studies (Escombe et al. 2007, Qian et al. 2010, Gilkeson et al. 2013) suggest that high ventilation rates are achievable, these may not have predominant or established airflow pathways in some cases. When combined with activity such as human movement, opening and closing doors, changes in buoyancy due to heat sources, and use of fans for comfort, these variations in airflow patterns may change the dispersion of airborne microorganisms and further confound surface contamination patterns. An observation and sampling study conducted on a multi‐bed respiratory ward (Hathway et al., 2013) found that airborne microorganisms and particles varied with the different activities on a ward, with increases during healthcare worker activity and personal care, but little change with visitors on the ward. Nevertheless, the approach presented here gives a realistic mechanism for exploring potential deposition at design stage and identifying design options that are likely to minimize contamination.

### Observational study

The first hand data accrued from the observational study is of fundamental importance for the modeling of human hand‐to‐surface contact events, allowing insight into the different types of care and how these also the behavior of nurses and doctors. These data then formed the basis of a stochastic model of HCW behavior as they moved from one surface to another and consequently forms the driving force behind the subsequent model for pathogen accretion. While the data are only from one hospital with a single‐bed ward environment, comparison with other studies suggests the data are realistic and representative of typical health care in other hospital environments. However, it should be acknowledged that in applying the model to other hospital wards, it may be necessary to make adjustments to the location of surfaces and contact patterns. For example, in the current study, the position of the patient charts and information in the single rooms was treated as a far‐bed surface based on the practice in the observation study, while in the four‐bed room, it was treated as near‐bed based on Smith et al.(2012). In other hospitals, the position of charts may vary depending on room design, local practices, and care needs. Similarly, in wards where care routines differ substantially from those considered here, it may be necessary to conduct a further observational study to create new hand‐to‐surface contact patterns that are more representative of the environment.

### Probabilistic model

The pathogen accretion model (PAM) was developed from the growing understanding of hand contamination from surface contacts. This model focuses on the physical process of accruing pathogens onto either the skin or gloved surfaces of HCWs’ hands as they perform episodes of standard patient care. The model provides a framework which allows for the quantitative comparison of hospital room design including single‐ vs. four‐bed accommodation by means of HCW hand contamination. Application of PAM to the scenario rooms showed that differences were not clear cut and the positioning of the infectious patient had most effect on the final results. The spatial deposition of particles is influenced by the location of the ventilation supply inlet relative to the source. Locating a susceptible patient closer to the supply air is likely to reduce the risk of environmental contamination due to bioaerosol release from a neighboring patient. Locating the infectious patient in a four‐bed room without an unobstructed air pathway from the bed to the ventilation outlet caused the highest level of surface contamination of all scenarios tested. These findings concur with experiments and simulations conducted in King et al. (2013).

It is crucial to highlight that this is a flexible framework model, built from the increasing understanding of the mechanics behind pathogen transmission; however, the results are dependent on several data inputs to the model which all have a degree of uncertainty. Initial validation was successful against the limited available data in the published literature, but it is acknowledged that full validation of the model is very challenging as the microbiome, ventilation design, physical layout, and personnel variation of every hospital is highly specific. Data for the surface contamination patterns and the HCW hand–surface contact patterns were derived from CFD models and the observational study, respectively, as part of the study. While this leads to a reasonable degree of confidence in these data in the particular scenarios examined, both parameters are influenced by a number of factors as outlined above. The other model elements relied on published data to provide suitable inputs. As far as possible, these were applied as distribution functions to account for variability; however, the influence of factors such as temperature, humidity, and surface porosity are not explicitly considered in the model and cannot be determined from the current results. With further data, it may be possible to include these factors, for example, into the transfer efficiency parameters.

It should also be emphasized that the current model does not simulate the pathogen accretion process over a particular time period. The CFD deposition pattern is used to generate a likely surface contamination pattern for a given room, and the PAM then simulates the contact sequence and likely resulting accretion. The resulting hand contamination is presented in the study as a normalized parameter to enable relative comparison between scenarios, as the model does not include transient factors that may influence the actual microbial concentration on HCW hands. In reality, microbial viability on surfaces and on hands will be a factor, and this is influenced by temperature and humidity as well as surface properties. Hand hygiene may also influence microbial viability; alcohol gels are known to exert residual microbiocidal effects due to remaining residue on HCWs’ hands (Bogusz et al., 2013) which would also influence the actual microbial concentrations. The aerosol release of microorganisms will also be a continuous and transient process, with surfaces never having a steady‐state concentration due to ongoing deposition interspersed with cleaning activities. Although this means that the model cannot explicitly model infection risk, the current formulation enables exploration of the likely importance of different design and care factors and incorporates the combined effects of airflows and human activities. In particular, it enables direct and quantitative comparison between different ward designs under the same care conditions. The framework also allows for increasingly precise specification of surface contamination levels, transfer parameters, and HCW contact patterns with input of further data from sampling and observation studies.

## Conclusions

The aim of this study was to provide a mathematical model which quantifies the relative contamination levels of healthcare workers’ hands from surfaces within hospital rooms. This is achieved through a multidisciplinary approach coupling CFD simulations with clinical observation of HCW movement and Markov chain Monte Carlo modeling of hand contamination. An observational study of patient care in a single‐bed hospital ward showed that hand hygiene choice and frequency varied strongly. HCWs performing short episodes of care had a predilection for alcohol rub. In other care types, the usage of alcohol rub or soap and water was 50/50. HCW surface contact patterns in rooms were modeled by a Markov chain and fed into a mathematical model to calculate the pathogen colonization level on hands after patient care.

A parametric study indicates that hand contamination levels depend highly on care type (*P* < 0.0001), the number of surface contacts (*P* < 0.001), and the distribution of surface pathogens (*P* = 0.05). Highest hand contamination was predicted during *Personal care* despite this having the highest levels of hand hygiene. This is due to the greater number of contacts on the more highly contaminated near‐bed surfaces. Increasing the ventilation rate is likely to have a small benefit on environmental contamination in a single‐bed room, while the relative location of infectious patients and ventilation supply and extract grilles had a significant effect in a four‐bed room (*P* = 0.05). Comparison of four patients housed in single rooms against a four‐bed room suggests contamination on the HCWs’ hands is likely to decrease monotonically after care in single rooms, but is likely to increase during contact with subsequent patients in four‐bed rooms. The results support the hypothesis that single‐patient rooms reduce the risk of HCAI, highlighting that this benefit may extend to risk of infection due to environmental contamination not just the more obvious airborne and direct contact transmission routes. The findings also suggest that ventilation design and room layout may affect environmental contamination and subsequent contact transmission risks in multi‐bed environments. This has implications for those designing wards as well as operational aspects in terms of the most suitable location for infectious or susceptible patients.

This study makes a significant contribution to the body of evidence that support the NHS in making decisions about hospital design and whether or not to incorporate single rooms.

## Conflict of interests

None to report.
